# RETRACTED: Shoaib et al. Neuroprotective Effects of Dried Tubers of *Aconitum napellus*. *Plants* 2020, *9*, 356

**DOI:** 10.3390/plants14243734

**Published:** 2025-12-08

**Authors:** Ambreen Shoaib, Hefazat Hussain Siddiqui, Rakesh Kumar Dixit, Sahabjada Siddiqui, Badrud Deen, Andleeb Khan, Salman H. Alrokayan, Haseeb A. Khan, Parvaiz Ahmad

**Affiliations:** 1 Department of Clinical Pharmacy, Faculty of Pharmacy, Jazan University, Jazan 45142, Saudi Arabia; amber8739@yahoo.com; 2 Department of Pharmacology, Faculty of Pharmacy, Integral University, Lucknow 226026, India; siddiquihefazat7@gmail.com; 3 Department of Pharmacology, King George Medical University, Lucknow 226003, India; dixitkumarrakesh@gmail.com; 4 Department of Biotechnology, Era’s Lucknow Medical College & Hospital, Era University, Lucknow 226003, India; sahabjadabiotech04@gmail.com; 5 Department of Pharmacology & Toxicology, Faculty of Pharmacy, Jazan University, Jazan 45142, Saudi Arabia; andleeb.tox@gmail.com; 6 Department of Biochemistry, College of Science, King Saud University, Riyadh 11451, Saudi Arabia; salrokayan@ksu.edu.sa (S.H.A.); khan_haseeb@yahoo.com (H.A.K.); 7 Botany and Microbiology Department, College of Science, King Saudi University, Riyadh 11451, Saudi Arabia

The journal retracts the article, “Neuroprotective Effects of Dried Tubers of *Aconitum napellus*” [1], cited above.

Following publication of a correction to this article [2], additional concerns were brought to the attention of the Editorial Office regarding the integrity of the image presented in this article [1].

Adhering to our standard procedure, an investigation was conducted by the Editorial Office and the Editorial Board, which confirmed an overlap between Figure 1 published in [1] and Figure 1 published in [3], showing different experimental conditions. While the authors collaborated within this process, raw material meeting the journal’s requirements for original images could not be provided for Editorial Board Evaluation. As a result, the Editorial Board has lost confidence in the reliability of the findings and decided to retract this publication [1], as per MDPI’s retraction policy (https://www.mdpi.com/ethics#_bookmark30).

This retraction was approved by the Editor-in-Chief of the *Plants* journal.

Ambreen Shoaib, Hefazat Hussain Siddiqui, Sahabjada Siddiqui, and Badrud Deen disagree with this retraction. The remaining authors did not provide a comment on this decision.
